# Indocyanine Green-Based Fluorescence-Guided Surgery in a Male Infant with Anorectal Malformation

**DOI:** 10.1055/s-0042-1750029

**Published:** 2022-08-23

**Authors:** Irene Paraboschi, Laura Privitera, Stavros Loukogeorgakis, Stefano Giuliani

**Affiliations:** 1 Wellcome/EPSRC Centre for Interventional & Surgical Sciences, University College London, London, United Kingdom; 2 Department of Specialist Neonatal and Pediatric Surgery, Great Ormond Street Hospital for Children NHS Foundation Trust, London, United Kingdom; 3 Cancer Section, Department of Developmental Biology and Cancer Programme, UCL Great Ormond Street Institute of Child Health, London, United Kingdom

**Keywords:** anorectal malformation, fluorescence-guided surgery, indocyanine green, children, real-time imaging

## Abstract

Reconstructive techniques for complex anorectal malformations (ARMs) require intestinal pull-through on vascular pedicles. Traditionally, the visual inspection of the intestinal perfusion is the sole modality adopted to assess tissue viability.

In this article, we report the case of a child with a rectourethral prostatic fistula, who had a Peña's descending colostomy with distal mucous fistula in the neonatal period and a posterior sagittal anorectoplasty at 6 months of life. The ARM repair was guided by indocyanine green (ICG), which was intravenously administered to evaluate the blood flow of the intestinal pull-through using the EleVision IR system (Medtronic Ltd, U.K.). ICG-based fluorescence-guided surgery helped to define the proximal resection margin, impacting intraoperative decision making, and no postoperative complications occurred.

We envisage that this technology will become part of the armory of pediatric surgeons soon, by reducing the risk of intra- and postoperative complications.

## Introduction

Reconstructive techniques for complex anorectal malformations (ARMs) require intestinal pull-throughs on vascular pedicles.

Traditionally, the visual intraoperative assessment of the intestinal perfusion has been the only technique adopted to evaluate the vascular blood supply.

More recently, however, fluorescence-guided surgery (FGS) has emerged as a promising intraoperative imaging modality that allows surgeons to visualize tissue vascular perfusion in real-time by administering near-infrared (NIR) fluorescent dyes or fluorescently labeled molecules.

Indocyanine green (ICG)-based FGS has proved to be a powerful tool to perform procedures in which the visual assessment may not be very reliable to determine good vascular perfusion to the tissue.

In particular, ICG, a U.S. Food and Drug Administration-approved water-soluble tricarbocyanine fluorophore with a well-established safety profile, is employed with increasing interest in several fields of pediatric surgery, 1 2 3 ranging from tumor resection to urogenital and plastic procedures.

However, its usefulness in pediatric gastrointestinal reconstructive procedures has been mostly anecdotal. 4 5

The aim of this case report is therefore to present the first step-by-step protocol to perform FGS in the repair of a male with ARM.

## Case Report



**Video 1** Intraoperative indocyanine green (ICG) fluorescence during the intestinal pull-through.


A 4-month-old male patient coming from a low-income country was referred to our tertiary referral hospital due to an ARM with a rectourethral prostatic fistula. He had a Peña's descending colostomy with distal mucous fistula in the neonatal period and underwent the posterior sagittal anorectoplasty (PSARP) at 6 months of age.

On the magnetic resonance imaging performed in his home country, it was difficult to appreciate the levator ani musculature, and both the right puborectalis muscle and the right pubococcygeus muscle appeared relatively smaller than the contralateral ones. The rectal pouch terminated at the level of S3 and was located at a right-sided position within the puborectalis sling.

**Fig. 1:**
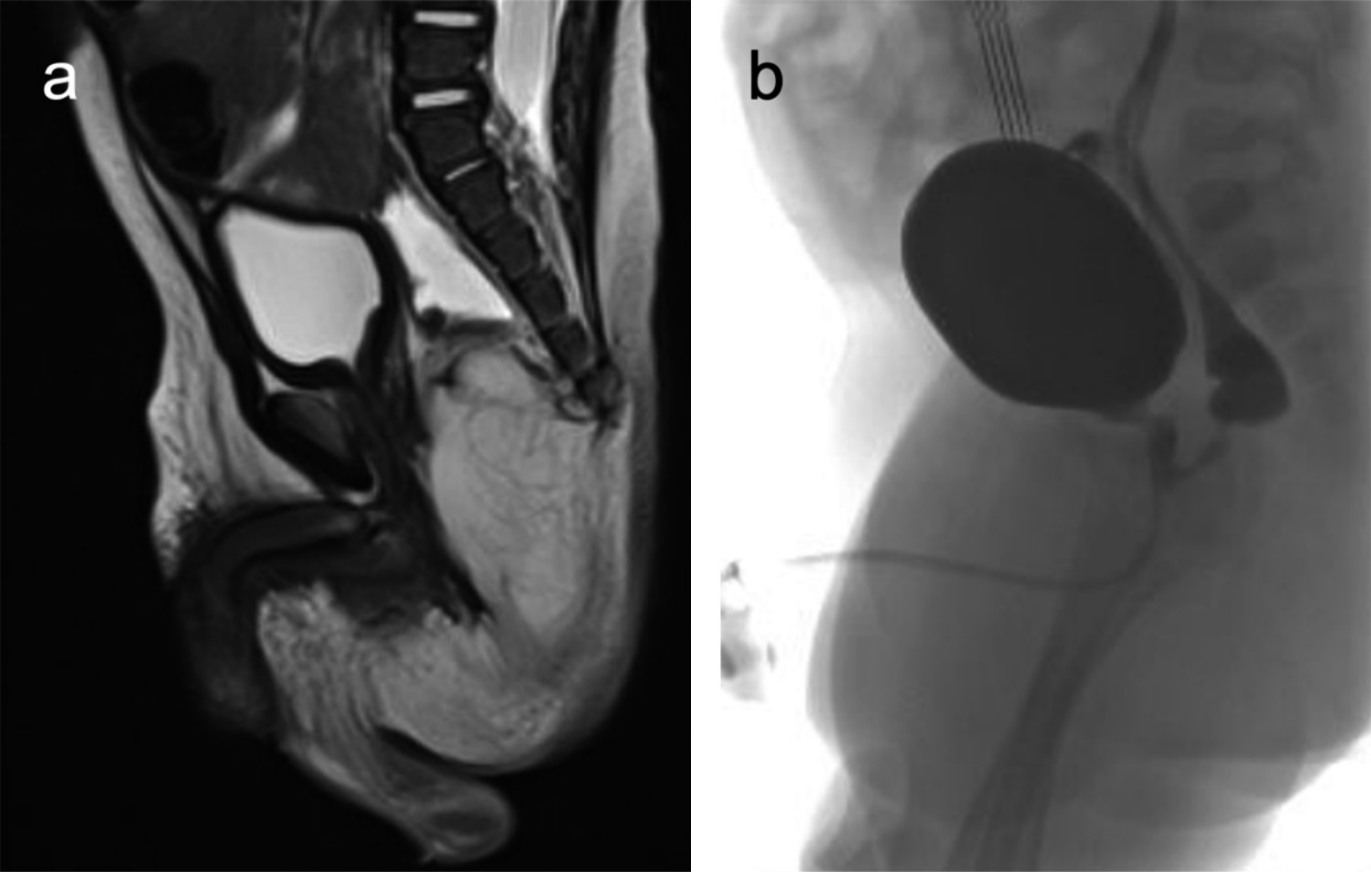
( **A** ) Preoperative magnetic resonance imaging (MRI) showing the anorectal malformation (ARM) with a rectourethral prostatic fistula. ( **B** ) Micturating cystourethrogram (MCUG) demonstrating a rectourethral prostatic fistula with a maximum diameter of 2 mm.

The ARM was associated with a fistula that traveled in the anteroinferior direction from the lower rectal pouch to the prostatic urethra ( Fig. 1A ).

This result was confirmed at the micturating cystourethrogram with the presence of a rectourethral fistula at the level of the prostate with a maximum diameter of 2 mm. The contrast passed through the fistula into the rectum, filling the entire distal loop. The rectal pouch terminated at the level of the tip of the coccyx just below the bladder base ( Fig. 1B ).

At the time of the PSARP, 1 mg (0.2 mg/kg) of ICG was intravenously administered to evaluate the vascular supply of the intestinal pull-through.

**Fig. 2:**
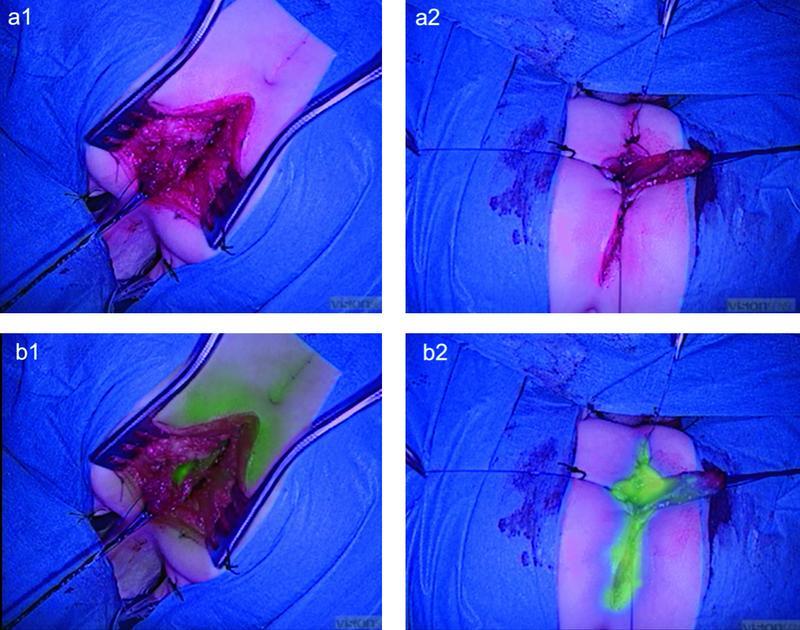
Intraoperative indocyanine green (ICG) fluorescence. ( **A1** , **A2** ) Visible light images. ( **B1** , **B2** ) Near-infrared (NIR) images. ( **B1** ) Assessment of intestinal perfusion during the pull-through lengthening maneuvers was a valuable first step after disconnecting the rectourethral fistula. ( **B2** ) A second dose of ICG was injected to visualize the vascular perfusion of the intestine and perineal wound edges before the anoplasty.

Favorable fluorescence was obtained within 1 minute using the EleVision IR system (Medtronic Ltd, U.K.) ( Fig. 2 ). The main vessel irrorating the distal end of the intestinal pull-through gained an intense fluorescent signal and maintained it for 2 minutes during the pull-through lengthening maneuvers, proving that the rectal vascular perfusion was adequate to reach the skin surface with minimal tension and no local ischemia ( Video 1 ).

The blood perfusion was considered adequate during the maneuvers to gain length for the pull-through to reach the skin surface with minimal tension.

After that, a second dose of ICG (1 mg, 0.2 mg/kg) was injected to visualize the vascular perfusion at the skin level and during the anoplasty ( Fig. 2 ). As always in this operation, we removed the two distal ends of the split distal rectum (total length: 20 mm) to reduce complications due to poor vascular perfusion.

For the first time, we were able to assess with accuracy the level of resection of the distal part of the rectum with the help of the fluorescent dye. Then, the anoplasty was performed between well-perfused bowel and perineal skin, using interrupted 5–0 PDS stitches (Ethicon, Johnson & Johnson Medical Devices Companies).

Milk feeds were started on the same day of the surgery. The postoperative course was favorable with the stoma being active on the first postoperative day. The baby was discharged home 6 days following surgery. No postoperative complications occurred either during the hospitalization or in the follow-up. Colostomy was closed 5 weeks after the PSARP and the baby was doing well at the 6-month follow-up, experiencing regular bowel movements.

## Discussion

The management of ARM has significantly improved after the introduction of the PSARP procedure by Peña and DeVries in the early 1980s. 6 This procedure soon achieved great recognition worldwide, and surgeons dealing with this complex malformation early recognized the tremendous progress provided by the technique in the management of this congenital anomaly.

Despite the growing experience and body of information globally, the treatment of ARMs continues to be a challenge for pediatric surgeons, and complications remain a significant problem. 7

In particular, anal stricture and dehiscence are frequent short-term postoperative complications following ARM repair. 8 Various reasons for their occurrence have been implicated, including inadequate blood supply, tension at the anastomosis, or damage of the intramural blood supply during close dissection on the rectal wall. 7

In recent years, ICG-based FGS has brought better intraoperative visualization for several pediatric operations. 1 2 3 ICG has unique pharmacokinetics: by binding albumin, IGC is normally confined in the vascular stream and entirely excreted into the biliary tract within a few hours after injection.

Therefore, ICG-based FGS has been adopted for improving the accuracy of biliary flow delineation during pediatric laparoscopic cholecystectomies and Kasai hepatoportoenterostomy. 1 More recently, this technology has also been employed to evaluate organ and tissue perfusion in children. 1 2 3 However, its role in guiding reconstructive gastrointestinal procedures in pediatrics has not been determined conclusively. 4 5

Adequate perfusion is a well-recognized requisite for good healing of an intestinal anastomosis during abdominal surgical procedures. Identifying an adequate vascular supply and maintaining a good vascular pedicle without tension or kink at the anastomosis site is crucial for good surgical outcomes. In the field of pediatric surgery, anatomic variations and congenital aberrations are extremely frequent and make the latter rather challenging.

Our report confirmed that ICG-based FGS is a safe and effective intraoperative imaging modality in the pediatric population, and no side effects were reported. We used the dose of 0.2 mg/kg (repeated twice; total dose 0.4 mg/kg) and obtained a high-quality fluorescence for 2 minutes without difficulties. The dose we used was within the range reported in the literature to date (0.05 to 0.5 mg/kg). 1 The perfusion of the intestinal pull-through could be determined after 1 minute following intravenous administration of ICG. Compared with clinical visual intraoperative assessment, ICG-based FGS provided objective data on tissue perfusion, and it impacted the intraoperative decision making confirming the proximal resection margin. ICG injection helped assess the perfusion of the pull-through bowel segment after separation of the fistula (to gain an adequate length), as well as the vascular supply of the distal intestine to decide where to start the anoplasty. Moreover, ICG-based FGS assisted to monitor the skin and mucosa perfusion at the edges of the anoplasty, aiming to reduce the risk of postoperative complications at this site.

Regarding optical imaging devices, several cameras have been employed in children to detect the fluorescence signal and guide surgical procedures in real-time. The Photodynamic Eye marketed by Hamamatsu Photonics Co (Hamamatsu, Japan) and the Image 1 S marketed by the Karl Storz GmbH & Co (Tuttlingen, Germany) have been the most used devices. The EleVision IR system allowed us to define the vascular supply of the intestinal pull-through and the anoplasty. This system has two independent channels for the visible and the NIR signals making the quality of the image excellent and very sensitive to small doses of ICG. This is a significant advantage in children, particularly when prone, as it is less likely to interfere with saturation probes. In fact, having absorption spectra used by optical-technology-based monitors, intravenously administered vital dyes, such as ICG, may result in misreading percutaneous oxygen saturation (SpO2) measured via pulse oximetry. 9 Interestingly, the EleVision IR platform (Medtronic Ltd) helped us keep the amount of the dye low enough to not cause any anomalies in the pulse oximeter. The other advantage of a dual-channel device is that we could keep the fluorescence image on for a long period of time. This was helpful during the pull-through lengthening and the formation of the anastomosis.

Even if more studies involving larger cohorts of patients and continued experience with fluorophores and optical imaging systems are needed in the repair of ARM, we envisage that FGS will soon become an integral part of the PSARP procedure. We believe this will reduce the rate of intraoperative complications and the rate of stricture and dehiscence of the neoanus.

## Conclusion

ICG fluorescence using the EleVision IR system (Medtronic Ltd) was safe and effective in intraoperatively evaluating the rectal blood supply with high definition and in real-time. We have described the key steps to achieve a useful FGS in the repair of a male with ARM.

## Notes

New Insights and Importance for the Pediatric Surgeon The employment of fluorescence-guided surgery (FGS) has been gaining popularity in many fields of paediatric surgery, where it has shown great potentials to improve both surgical and oncological outcomes while minimizing anaesthetic time and lowering healthcare costs. However, its role in guiding reconstructive gastrointestinal procedures in paediatric is still at the beginning. Here, we present the first step by step protocol to perform FGS during a posterior sagittal anorectoplasty (PSARP) in a child with an Anorectal malformation (ARM) with a recto-urethral fistula.
